# Selection of mesophotic habitats by *Oculina patagonica* in the Eastern Mediterranean Sea following global warming

**DOI:** 10.1038/s41598-021-97447-5

**Published:** 2021-09-13

**Authors:** Stephane Martinez, Jessica Bellworthy, Christine Ferrier-Pagès, Tali Mass

**Affiliations:** 1 Department of Marine Biology, Leon H. Charney School of Marine Sciences, University of Haifa, Haifa, Israel; 2 Morris Kahn Marine Research Station, The Leon H. Charney School of Marine Sciences, University of Haifa, Sdot Yam, Israel; 3 Coral Ecophysiology Team, Centre Scientifique de Monaco, 8 Quai Antoine 1er, Monaco City, 98000 Monaco; 4 The Interuniversity Institute of Marine Sciences, Eilat, Israel

**Keywords:** Ecology, Evolution, Physiology, Zoology, Climate sciences, Ecology, Environmental sciences

## Abstract

Globally, species are migrating in an attempt to track optimal isotherms as climate change increasingly warms existing habitats. Stony corals are severely threatened by anthropogenic warming, which has resulted in repeated mass bleaching and mortality events. Since corals are sessile as adults and with a relatively old age of sexual maturity, they are slow to latitudinally migrate, but corals may also migrate vertically to deeper, cooler reefs. Herein we describe vertical migration of the Mediterranean coral *Oculina patagonica* from less than 10 m depth to > 30 m. We suggest that this range shift is a response to rapidly warming sea surface temperatures on the Israeli Mediterranean coastline. In contrast to the vast latitudinal distance required to track temperature change, this species has migrated deeper where summer water temperatures are up to 2 °C cooler. Comparisons of physiology, morphology, trophic position, symbiont type, and photochemistry between deep and shallow conspecifics revealed only a few depth-specific differences. At this study site, shallow colonies typically inhabit low light environments (caves, crevices) and have a facultative relationship with photosymbionts. We suggest that this existing phenotype aided colonization of the mesophotic zone. This observation highlights the potential for other marine species to vertically migrate.

## Introduction

As a result of increasing global temperature, multiple species have responded with conspicuous changes in phenology e.g., earlier onset of annual flowering in plants^1^ and range shifts towards higher latitudes and altitudes^2^. A meta-analysis revealed a median rate of poleward migration of 16.9 km per decade for terrestrial species, but high inter-species variation in migration rate and the ability to track isotherms^2^. For example, less mobile species change their distribution more slowly; sessile animals and plants can only migrate through reproduction and seed dispersal. Alternatively, a suitable substitute environment may exist nearby, such as the poleward facing, cooler side of a mountain, reducing the need for individuals to latitudinally track temperature change^3^. Marine taxa show comparable or greater range shifts as terrestrial species with the fastest range expansions observed in phytoplankton (469.9 ± 115.3 km per decade), which have short generation times and are advected with moving water masses^4^.

Among marine species, scleractinian corals, which live in symbiosis with dinoflagellates belonging to the family Symbiodiniaceae^5^, are increasingly one of the most severely threatened by climate change^6,7^. The dominant threat to symbiotic corals is warming sea surface temperature, which can induce the loss of symbionts and/or associated photosynthetic pigments, a phenomenon called bleaching^6,8–10^. As symbiont photosynthetic products cover most of the energetic needs of the animal host, bleaching can ultimately lead to coral death^10^. Similar to other organisms, the potential for coral populations to migrate to higher, cooler latitudes was suggested. Authors of a meta-analysis concluded that an average poleward migration of tropical corals was observed between 1974 and 2012 as recruit numbers declined by 85% in the tropics, concurrent to a 78% recruitment increase at latitudes > 20° over the same period^11^. In the Mediterranean Sea, a northward and westward range expansion is observed for scleractinian corals such as *Oculina patagonica* along the Spanish coasts^12–14^. However, in comparison to the aforementioned rapidly moving phytoplankton, corals have longer generation times, greater age of sexual maturity, and are sessile as adults; all of which impose limits on the rate of migration. An alternative to horizontal or latitudinal migration may be vertical migration to deeper, cooler waters, requiring reduced absolute distance to move. Deeper corals are less likely to experience thermal anomalies^15,16^. For example, the Mediterranean scleractinian coral *Cladocora caespitosa* has lower rates of thermal induced mortality at depth compared to the shallows, leading researchers to predict changes in the depth distribution of this population^6^. However, lack of connectivity between deep and shallow populations^17^, specific environmental adaptation^18,19^, and reduced reproductive output of deeper colonies^20,21^ may place limits on the migratory potential and viability of migrants^22^. In addition, symbiont net photosynthesis rates are reduced at mesophotic depths^23,24^, thus, corals need to shift from an autotrophic, symbiont-based mode of nutrition to heterotrophic prey-capture to cover energetic needs^23,25–27^. Not all coral species are able to compensate for the reduced autotrophic energy input with the other nutritional modes, placing another potential caveat for the migration of corals to deeper waters. In light of these challenges, to date, there are scant observations of coral species migrating from shallow to deeper environments.

**Figure 1:**
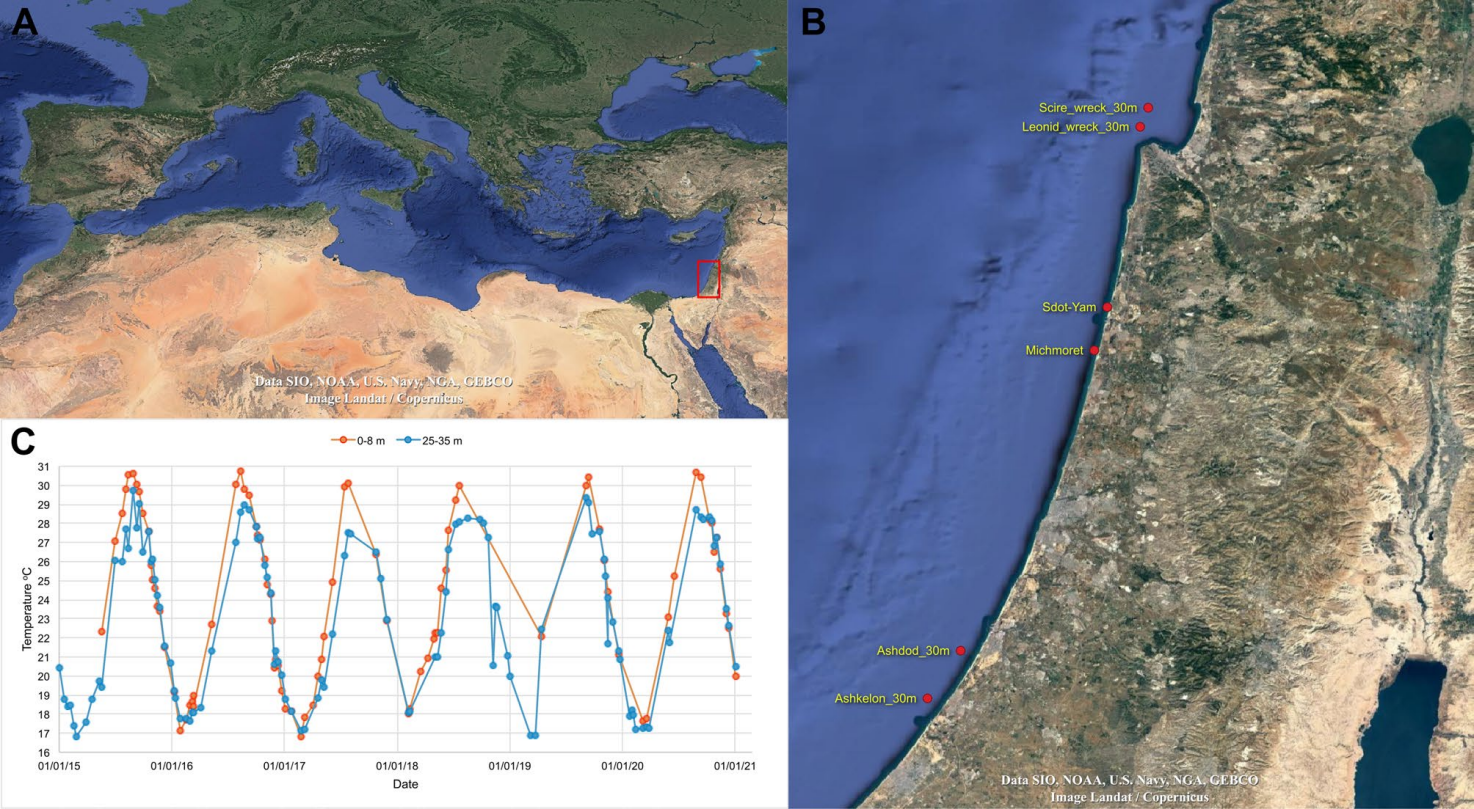
Sampling locations and records of *Oculina patagonica* at depths beyond 25 m and sampling locations in the present study, shallow: Sdot Yam, deep: Leonid wreck. The graph shows the water temperature record sampled 1–2 times per month in front of the Israeli School of Marine Sciences in Michmoret at depths of 30 and 7 m. Data available: http://reco.ruppin.ac.il/eng/ satellite image by Google.

Unlike tropical corals where photosynthesis is obligatory, temperate coral species, such as those thriving in the Mediterranean Sea, may display seasonal bleaching or have high intraspecific plasticity in response to light intensity with conspecifics found both exposed to light with symbionts and in the dark with few symbionts^23,27–30^. One such species is *Oculina patagonica*^31^, a stony coral from the taxonomic family Oculinidae. It inhabits coastal waters of the Mediterranean Sea, under a wide range of environmental conditions^14,32,33^. In the northwest Mediterranean, this species thrives under high light and a temperature range of 12–24 °C^14^. In the southeast Mediterranean, *O. patagonica* can be found in both high and low light environments and temperatures of 16–31 °C^7^. Relatively low mortality rates, early sexual maturity (1–2 years), rapid growth (1–2 cm year^−1^), and plasticity in its association with photosymbionts make this an opportunistic coral with the ability to rapidly colonize new environments^14,30,34,35^. Despite such plasticity, *O. patagonica* has never been reported below 15 m depth. All publications to date report a peak in its abundance at depths between 3 and 6 m and sharp declines below 9 m depth^14,32,33^. However, surveys conducted along the Israeli coasts by the Morris Kahn Marine Research Station Long Term Ecological Research program (MKMRS LTER; established in 2014—https://med-lter.haifa.ac.il/index.php/en/data-base) recently reported the presence of *O. patagonica* much deeper than usual, at 30 m depth (Ashdod, Israel, autumn 2019). One year later, in autumn 2020, colonies were also recorded at a second monitoring site approximately 20 km south (Ashkelon, Israel), and approximately 150 km north (Scirè and Leonid wrecks, Haifa Bay, Israel. We suggest that increasing sea surface temperature over the last three decades in the eastern Mediterranean (> 3 °C)^36^ has breached the upper thermal threshold of *O. patagonica*, and concomitant with warming in deeper waters has drawn this species to seek refuge in the cooler, deeper waters along the Israeli coast (1–2 °C cooler in the summer, Fig. 1C). Although these colonies may have been present yet undetected at deeper sites before 2019, mesophotic *O. patagonica* has never been reported in the literature^14,32,33^.

This study aimed to sample colonies from both shallow and deep sites in order to compare physiology, morphology, and trophic position (TP). We sought to understand what elements of this coral's ecology permitted vertical migration and how the newly established deeper population has acclimated to the new environment. These analyses are one of the few records of coral migration from shallow to deep water, adding understanding for the applicability of the Deep Reef Refugia Hypothesis^10,22^ in the face of environmental change.

## Results

### Photochemistry, physiology, and calcification rate

**Figure 2:**
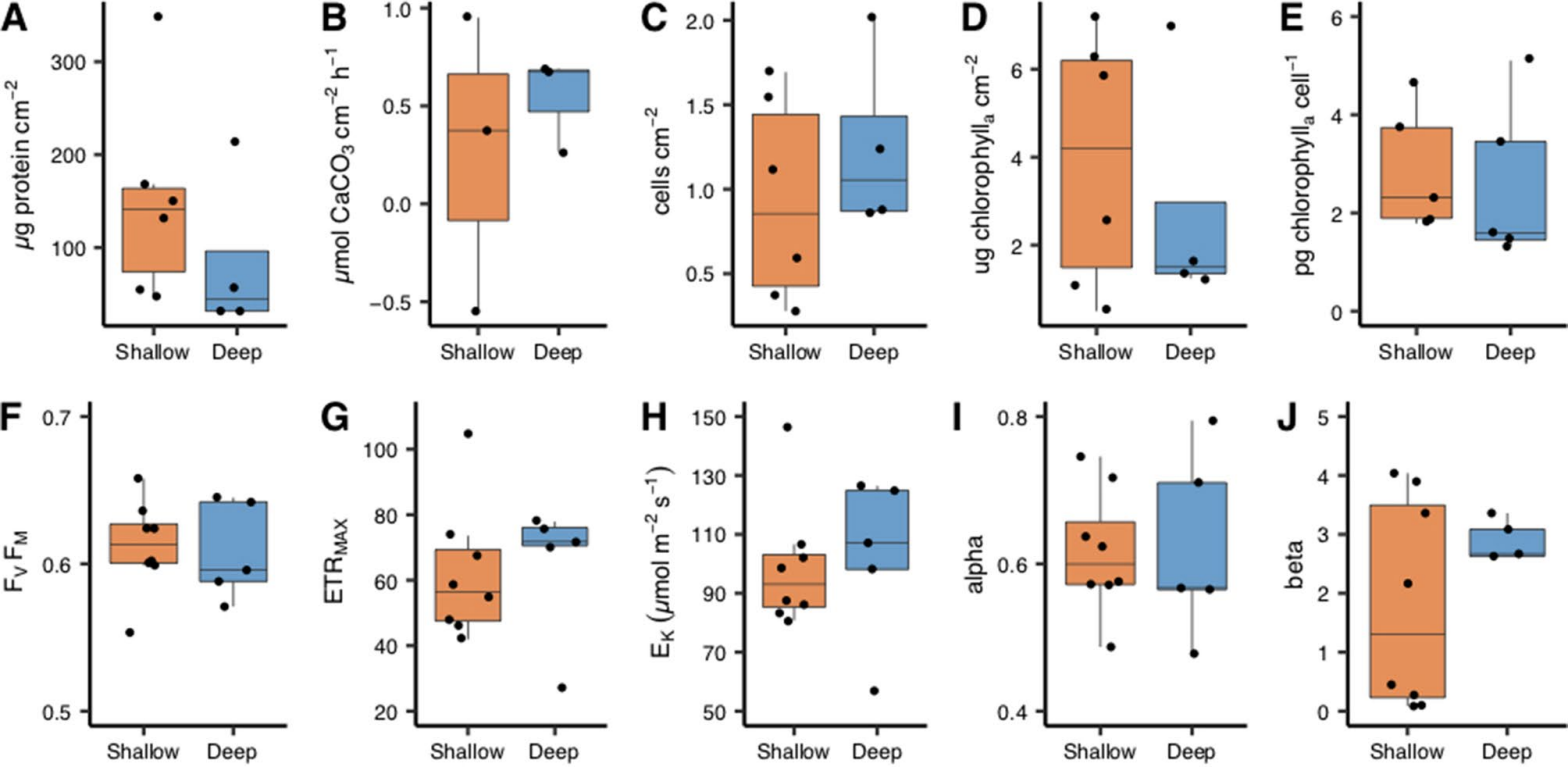
Physiological and photochemical analyses of *Oculina patagonica* from 30 m ('Deep', blue boxes) and 2 m ('Shallow', red boxes) water depth. Subplots in upper panel are (**A**) protein concentration, (**B**) calcification rate, (**C**) algal cell density normalized to protein concentration, (**D**) chlorophyll concentration per skeletal surface area, (**E**) chlorophyll concentration per algal cell. Lower panel (**F**) maximum quantum yield, (**G**) relative maximum electron transport rate, (**H**) saturating irradiance (µmol m^−2^ s^−1^), (**I**) photosynthetic efficiency at light limiting irradiances, (**J**) the downward slope following maximal electron transport rate. Horizontal black lines within boxes are median values and box limits represent 1st and 3rd quartiles. Whiskers represent 1.5 times the interquartile range. Round black points are individual sample data.

There were no significant depth-dependent differences found in all physiological and photochemical parameters assessed (Fig. 2, Table 1): calcification rate, chlorophyll *a* cm^−2^, symbiont cells normalized to surface area or host protein concentration, protein concentration cm^−2^, F_V_/F_M_, rETR_MAX_, α, β, and E_K_. Statistical differences were inferred with a Student's T-test except for the latter two parameters where a non-parametric Kruskal–Wallis test was used.

**Table 1:** Results of statistical analyses comparing characteristics of *Oculina patagonica* coral colonies originating from shallow vs. deep collection sites.

Parameter	Method	Test statistic	d.f.	*p* value
**Coral physiology**				
Calcification rate	T-test	t = − 0.620	2.406	> 0.05
Chlorophyll cm^−2^	T-test	t = 0.607	6.733	> 0.05
Cells cm^−2^	Kruskal–Wallis	chi^2^ = 0.045	1	> 0.05
Chlorophyll cell^−1^	Kruskal–Wallis	chi^2^ = 1.633	1	> 0.05
Protein cm^−2^	T-test	t = − 1.065	7.559	> 0.05
**Photophysiology**				
F_V_/F_M_	T-test	t = − 0.201	8.202	> 0.05
Alpha/α	T-test	t = 0.108	6.237	> 0.05
Beta/β	Kruskal–Wallis	chi^2^ = 0	1	> 0.05
*r*ETR_MAX_	T-test	t = 1.639	7.775	> 0.05
E_K_	Kruskal–Wallis	chi^2^ = 0.536	1	> 0.05
**Morphology**				
Distance between septa	Kruskal–Wallis	chi^2^ = 21.369	1	< 0.00001***
Distance between cenosarc spines	Kruskal–Wallis	chi^2^ = 3.945	1	0.047*
Septa diameter	T-test	t = − 0.770	71.153	> 0.05
Calyx diameter	Kruskal–Wallis	chi^2^ = 0.0003	1	> 0.05
Distance between calyxes	Kruskal–Wallis	chi^2^ = 0.434	1	> 0.05
**C:N assimilation**				
Host dissolved δ^13^C	T-test	t = − 0.050	3.151	> 0.05
Symbiont dissolved δ^13^C	T-test	t = 0.782	4.382	> 0.05
Host particulate δ^13^C	T-test	t = − 12.184	2.917	0.001361***
Symbiont particulate δ^13^C	T-test	t = − 6.725	2.100	0.01884*
Host dissolved δ^15^N	T-test	t = 0.409	2.874	> 0.05
Symbiont dissolved δ^15^N	Kruskal–Wallis	chi^2^ = 1.089	1	> 0.05
Host particulate δ^15^N	T-test	t = − 4.338	6.000	0.004884***
Symbiont particulate δ^15^N	Kruskal–Wallis	chi^2^ = 5.333	1	0.02092*
Dissolved δ^13^C host vs. symbiont shallow	T-test	t = − 3.210	2.052	> 0.05
Particulate δ^13^C host vs. symbiont shallow	T-test	t = − 3.516	2.253	> 0.05
Dissolved δ^15^N host vs. symbiont shallow	T-test	t = − 1.069	3.999	> 0.05
Particulate δ^15^N host vs. symbiont shallow	T-test	t = − 0.231	5.988	> 0.05
Dissolved δ^13^C host vs. symbiont deep	T-test	t = − 5.368	4.049	0.005617**
Particulate δ^13^C host vs. symbiont deep	T-test	t = − 3.135	5.642	0.02194*
Dissolved δ^15^N host vs. symbiont deep	T-test	t = − 1.132	5.079	> 0.05
Particulate δ^15^N host vs. symbiont deep	T-test	t = 0.174	5.897	> 0.05
**Trophic position**				
Host	T-test	t = 0.252	6.52	> 0.05
Symbiont	T-test	t = − 2.32	5.97	> 0.05
Host vs. symbiont shallow	T-test	t = 1.72	3.45	> 0.05
Host vs. symbiont deep	T-test	t = 3.26	4.66	0.0249*
**Amino acids**				
Host valine δ^13^C	T-test	t = − 0.951	6.979	> 0.05
Host leucine δ^13^C	T-test	t = − 7.389	6.826	< 0.0002***
Host isoleucine δ^13^C	T-test	t = − 0.092	6.914	> 0.05
Host methionine δ^13^C	T-test	t = − 1.004	6.969	> 0.05
Host phenylalanine δ^13^C	T-test	t = − 1.749	6.313	> 0.05
Host glutamic acid δ^15^N	Kruskal–Wallis	chi^2^ = 5.333	1	> 0.05
Host phenylalanine δ^15^N	T-test	t = − 2.957	4.611	0.03493*
Symbiont valine δ^13^C	T-test	t = − 0.217	4.571	> 0.05
Symbiont leucine δ^13^C	T-test	t = − 2.026	4.810	> 0.05
Symbiont isoleucine δ^13^C	T-test	t = 0.044	5.923	> 0.05
Symbiont methionine δ^13^C	T-test	t = − 0.158	3.359	> 0.05
Symbiont phenylalanine δ^13^C	T-test	t = − 0.875	5.998	> 0.05
Symbiont glutamic acid δ^15^N	T-test	t = − 1.747	5.994	> 0.05
Symbiont phenylalanine δ^15^N	Kruskal–Wallis	chi^2^ = 4.083	1	0.04331*

Comparisons are considered statistically significant where p ≤ 0.05 and are displayed in bold type.

Asterisks indicate the level of significance *p < 0.05, **p < 0.01, ***p < 0.005. Degrees of freedom (d.f.).

### Species identification and morphology

**Figure 3:**
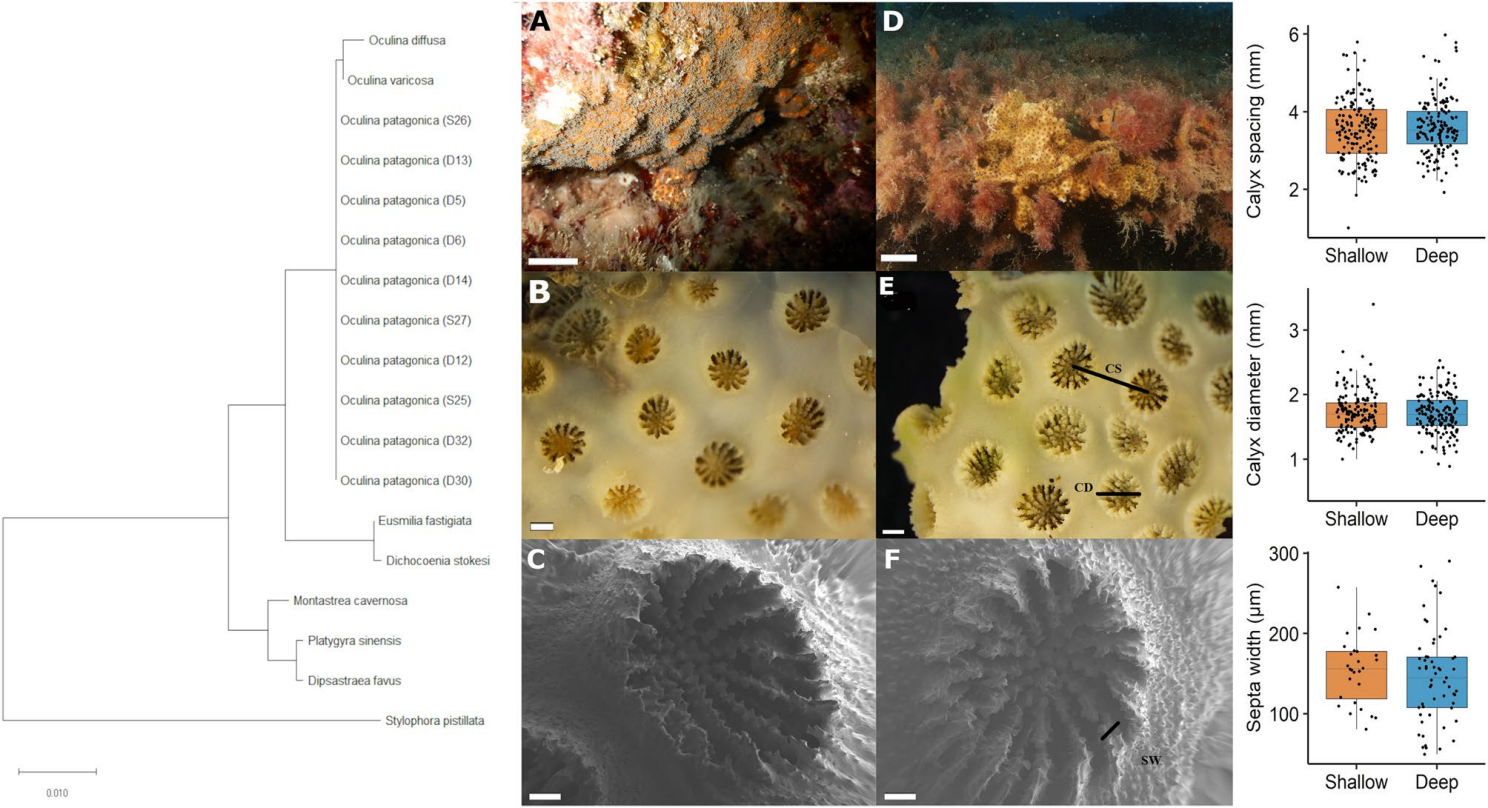
Verification of species identity of all colonies collected as *Oculina patagonica*, using both genomic (CO1 gene sequencing) and morphological features. The evolutionary history was inferred using the Neighbour-Joining method and evolutionary distances were computed using the Maximum Composite Likelihood method (units: number of base substitutions per site) using the software MEGA X. Images (**A**–**C**) are of shallow colonies and (**D**–**F**) deep colonies. *CS* Calyx spacing, *CD* Calyx diameter, *SW* Septa width. Scales bar lengths are 1 cm (**A**, **D**, in situ), 1 mm (**B**, **E**, binocular microscopy), and 100 μM (**C**, **F**, scanning electron microscopy, mag. × 60). Graphs in the right panel display measured skeletal features on *O. patagonica* from 30 m ('Deep', blue boxes) and 2 m ('Shallow', red boxes) water depth. Horizontal black lines within boxes are median values and box limits represent 1st and 3rd quartiles. Whiskers represent 1.5 times the interquartile range. Round black points are individual sample data. No statistically significant differences were present between deep and shallow corals (Student’s t-test, p ≥ 0.05).

The dominant ITS2 symbiont type was identified as *Breviolum psygmophilum* (synonym of *Symbiodinuium psygmophiulm*, clade B2) in sequenced samples from both collections depths. Two samples, one from shallow and one deep, hosted predominantly clade B2d (no associated species) at 68% and 56% relative abundance, respectively, though also maintained a less abundant population of *S. psygmophilum*. Coral host COI sequences of all samples shared 100% base pair similarity (Fig. 3). The consensus sequence had a 100% match to *Oculina patagonica* mitochondrial partial COI gene in NCBI nucleotide Blast (accession number: LN614380.1)^37^. Microscopic examination of skeletal morphology further verified that all samples belong to the species *Oculina patagonia*^38^. Specifically, all colonies were encrusting distinguishing the species from others in this genus that form either thin (e.g., *O. diffusa, O. valenciennesi, O. varicosa*) or robust branches (i.e., *O. rubusta*). Deep colonies had a tendency to form short, clumped vertical projections. Corallites were "crowded… (with) neat round exert walls''^38^ and measured between 1.84 and 5.98 mm in diameter (mean = 3.56 mm). Septa alternated between long and short. Costae were poorly developed without the appearance of conspicuous lines over the cenosteum, which appeared smooth when viewed under a binocular microscope (Fig. 3). Sampled colonies ranged from 3.7 to 12.7 cm (mean = 7.3 cm) at their widest diameter and typically formed elongate or elliptical colony shapes. Distances between polyps, polyp diameter, and the diameter of septa (Fig. 3) were not significantly different between sampling depths. The distance between septa was significantly smaller on shallow colonies (Kruskal–Wallis test, chi-squared = 21.369, df = 1, p-value = 3.788e^−06^) and the cenosarc spines were significantly further apart on shallow colonies (Kruskal–Wallis chi-squared = 3.945, df = 1, p-value = 0.047).

### Isotope analysis

**Figure 4:**
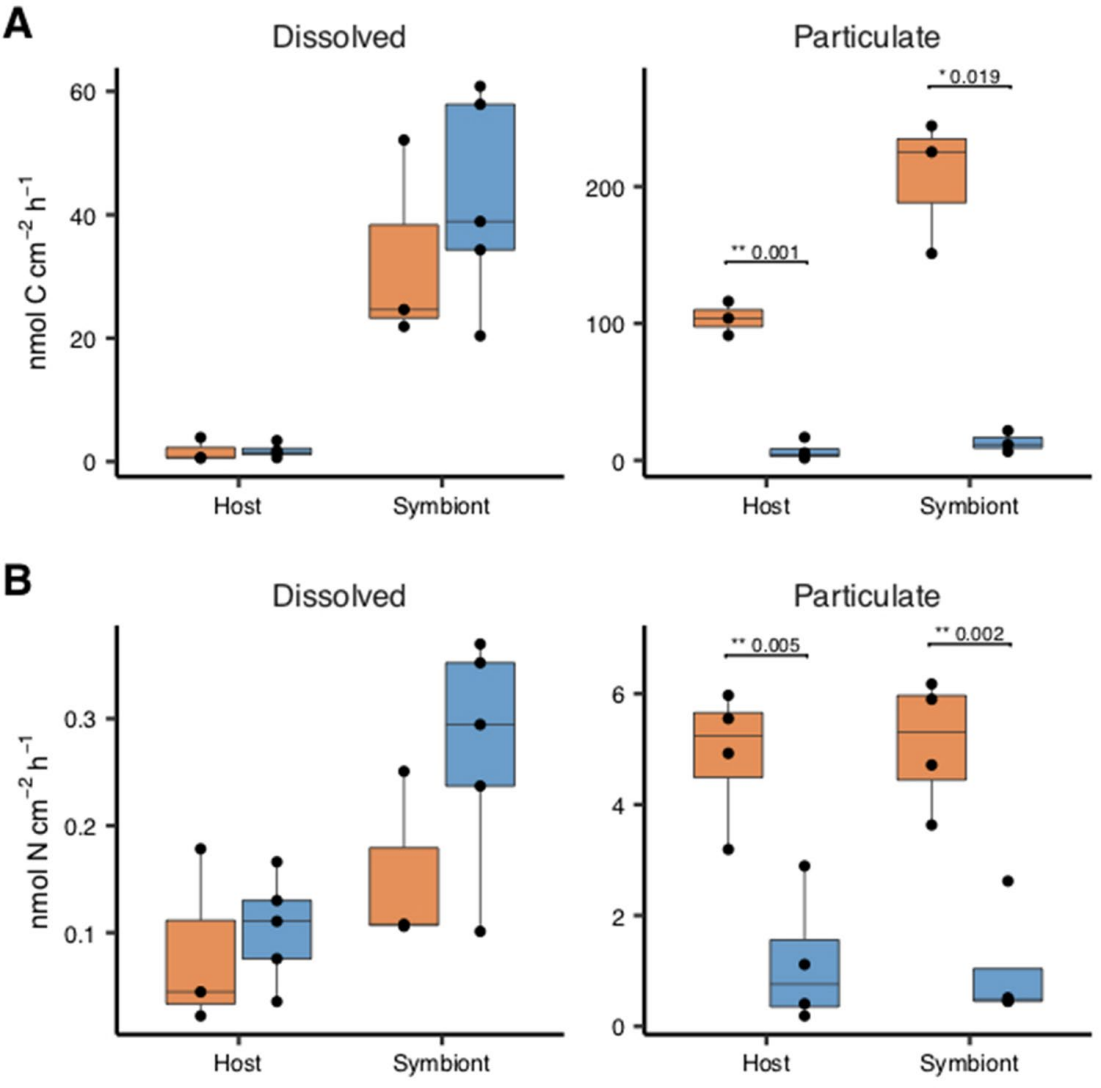
Carbon (top panel) and nitrogen (bottom panel) assimilation rates in the coral host tissue and algal symbionts via heterotrophy. Colonies originate from 30 m ('Deep', blue boxes) and 2 m ('Shallow', red boxes) water depth. Horizontal black lines within boxes are median values and box limits represent 1st and 3rd quartiles. Whiskers represent 1.5 times the interquartile range. Round black points are individual sample data. No statistically significant differences were present between deep and shallow corals in the dissolved fraction (Student’s t-test, p ≥ 0.05) but differences found in the uptake of particulate matter are displayed.

The rate of uptake of dissolved inorganic carbon and nitrogen was not significantly different between depths (Fig. 4). Algal symbionts in deep corals assimilated significantly more dissolved inorganic carbon (T-test, t = − 5.368, df = 4.049, p-value = 0.0056) and incorporated particulate carbon metabolised by the host (T-test, t = − 3.135, df = 5.642, p-value = 0.0219) compared to coral host incorporation. No such difference was observed in the assimilation of nitrogen. The incorporation rates of labelled particulate carbon and nitrogen were significantly higher in both host and symbiont tissues in shallow compared to deep corals (Table 1).

### Compound specific isotope analysis

**Figure 5:**
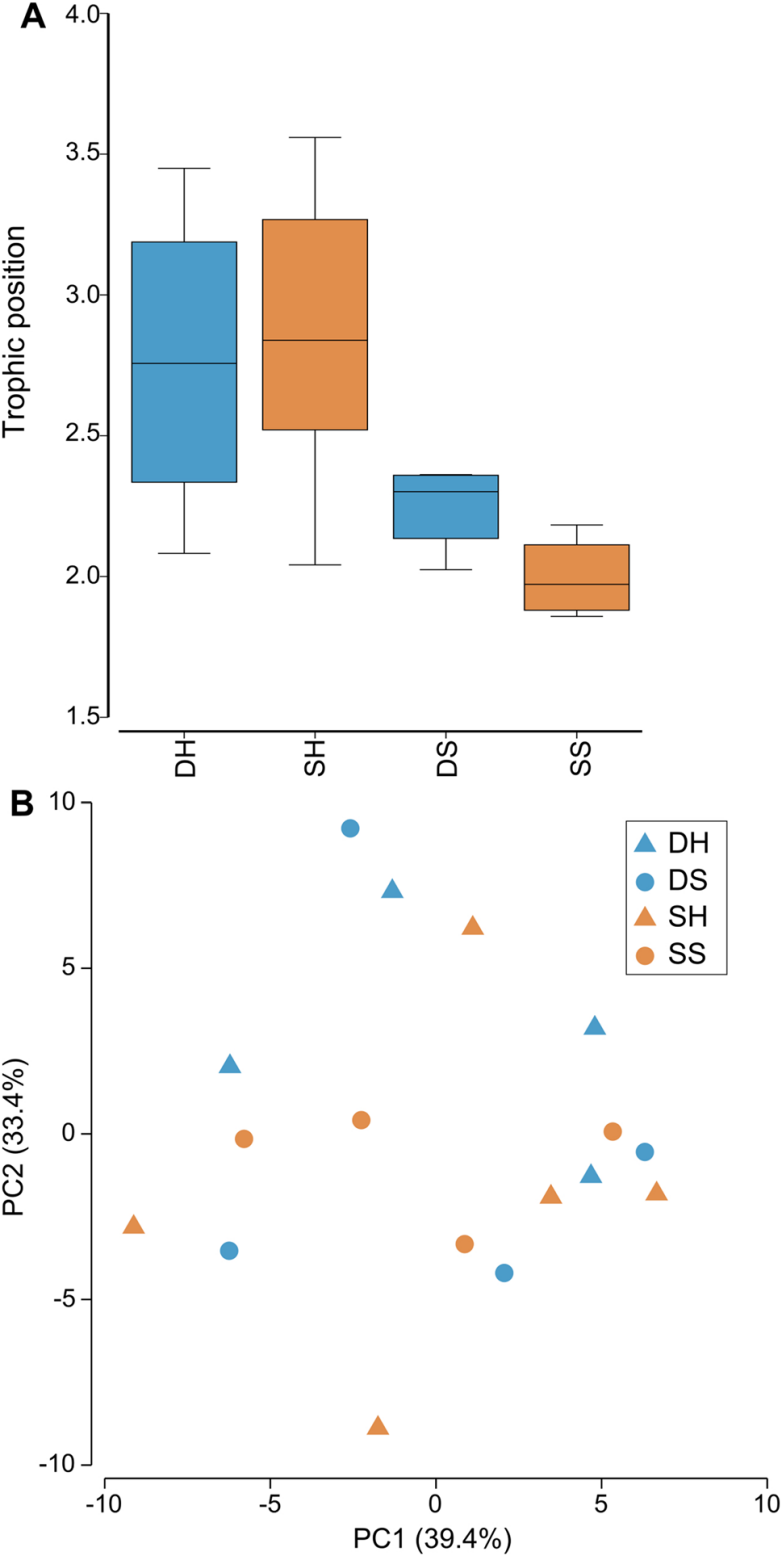
Calculated trophic position and carbon source of *Oculina patagonica* from 30 m ('Deep', blue) and 2 m ('Shallow', red) water depth. Sample are DH (deep host), DS (deep symbiont), SH (shallow host) and SS (shallow symbiont) (**A**) Calculated trophic position based on glutamic acid and phenylalanine nitrogen isotope. Horizontal black lines within boxes are median values and box limits represent 1st and 3rd quartiles. Whiskers represent 1.5 times the interquartile range. Round black points are individual sample data. (**B**) Carbon PCA of five essential amino acids (valine, leucine, isoleucine, methionine, and phenylalanine).

**Figure 6:**
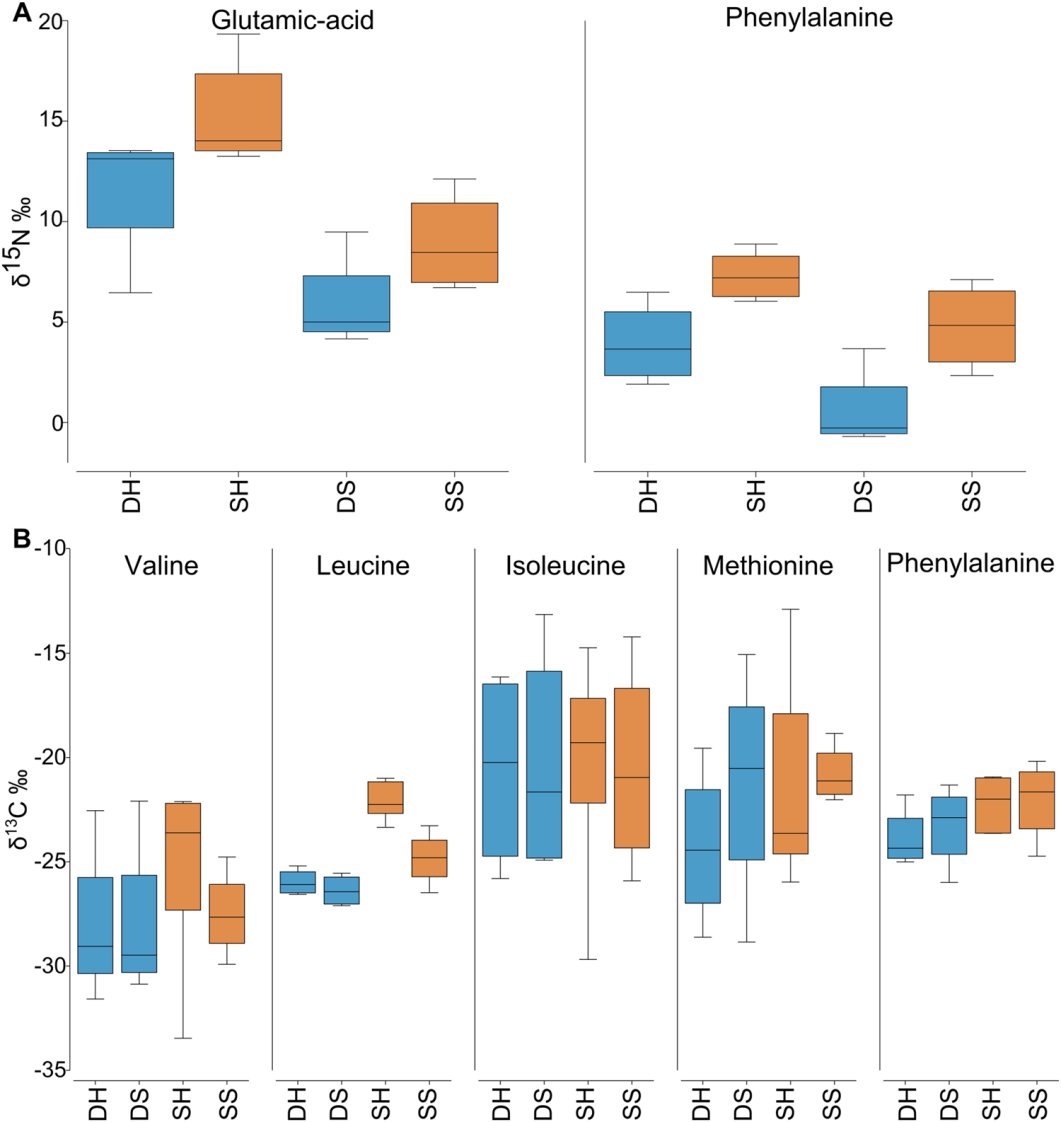
Amino acids isotopic value of *Oculina patagonica* from 30 m ('Deep', blue) and 2 m ('Shallow', red) water depth. Sample are DH (deep host), DS (deep symbiont), SH (shallow host) and SS (shallow symbiont). (**A**) Nitrogen isotopic value of two amino acids (glutamic-acid and phenylalanine) that are used for the calculation of the trophic position. (**B**) Carbon isotopic value of five essential amino acids (valine, leucine, isoleucine, methionine, and phenylalanine).

The δ^13^C of five essential amino acids (valine, leucine, isoleucine, methionine, and phenylalanine) was used to create the principal component analysis (Fig. 5B). ANOSIM analysis indicates an overall similarity between shallow and deep corals (p-value = 0.495) as well as between host and symbionts (p-value = 0.939). However, there was a significant difference in leucine δ^13^C between shallow and deep colonies host (T-test, t = − 7.389, df = 6.826, p-value = 0.0002) (Fig. 6B, Table 1). In addition, the δ^15^N values of phenylalanine used to calculate the trophic position (TP) were significantly higher in both host and symbionts (Host: T-test, t = − 2.957, df = 4.611, p-value = 0.0349; Symbiont: Kruskal–Wallis test, chi-squared = 4.083, df = 1, p-value = 0.0433) (Fig. 6A, Table 1), suggesting that the food source is different between the two depths.

The coral host TP in deep corals was calculated as 2.76 ± 0.58 and 2.87 ± 0.57 in shallow corals. Symbionts in deep corals had calculated TP’s of 2.25 ± 0.16 and shallow symbionts were 2.00 ± 0.15. Statistical analyses reveal no significant difference in either host or symbiont TP between depths (Host: T-test, t = 0.252, df = 6.52, p-value > 0.05; Symbiont: T-test, t = − 2.32, df = 5.97, p-value > 0.05; Fig. 5A, Table 1).

## Discussion

Populations of *O. patagonica* have been studied for over 25 years. Despite detailed ecological surveys, this species was never reported below 15 m depth^14,32,33^ until living colonies were sighted by the MKMRS LTER in 2019, in Ashdod, Israel. Our genomic and morphological data show as far as presently possible that all colonies collected from both deep and shallow sites belong to the reef-building species *O. patagonica*. Although the origin of these colonies remains to be proven (i.e., novel vertical migration from shallow populations or possible expansion of previously cryptic individuals already existing at this depth), evidently *O. patagonica* is increasing in abundance at mesophotic depths. When comparing shallow and deep colonies, we observed limited differences in terms of corals' physiology, TP, symbiont photophysiology, and dominant symbiont species. We thus suggest that prior adaptation to low light environments enabled *O. patagonica* to grow at mesophotic depths in the eastern Mediterranean Sea. In contrast, abundant literature documents depth-specific physiology and morphology for various Mediterranean^6,19,23^ and tropical coral species^19,39–41^ including depth-dependent adjustment of photosymbiont species^42,43^ or even symbiont genus^44,45^. However, in the present study, colonies of *O. patagonica* from both depths hosted *Breviolum psygmophilum*, as previously isolated from shallow *O. patagonica* and dominant in Mediterranean corals^46,47^.

The lack of depth dependant physiological differences likely results from the comparable light intensities at both collection sites (2–10 µmol photons m^−2^ s^−1^); matching light levels reduce the adaptation pressure to form a depth specific phenotype. Reef complexity creates distinct microenvironments^48^ such as the low light conditions under the overhangs from which *O. patagonica* resides at our shallow collection site. At both depths, colony physiology was more typical of mesophotic corals^6,24,49^ and had relatively different physiology compared to a temperate congener, *O. arbuscula*^50^ or the symbiotic stony coral *C. caespitosa*^23,51^ such as high chlorophyll concentration (3.9 ± 0.38 pg chlorophyll cell^−1^, an average of all corals combined), high photosynthetic efficiency (0.61 ± 0.002), low saturation irradiance (100.35 ± µmol m^−2^ s^−1^), and low calcification rates (0.402 ± 0.09 µmol CaCO_3_ cm^−2^ h^−1^). Overall, such physiology permits *O. patagonica’s* growth in low light environments and may in part explain the ability of this species to proliferate at mesophotic depths.

Our compound specific isotope analysis indicates that both shallow and mesophotic colonies were dominantly heterotrophic (coral host TP: deep = 2.76 ± 0.58, shallow = 2.87 ± 0.57), relying less on photoassimilates compared to other symbiotic corals, whose trophic index is typically closer to autotrophic organisms (TP ~ 1) e.g.,^52,53^. The coral tissue of *O. patagonica* had a TP higher than that of *Stylophora pistillata* and *Montipora capitata* grown in the dark for a month^53,54^ and a similar TP to the aposymbiotic Mediterranean coral *Melithaea erythraea*^55^. This high TP also contrasts with previous studies on the trophic ecology of *O. patagonica* from the Northern Mediterranean, which relies on the translocation of photoassimilates for its energetic needs^56^ or of *Cladocora caespitosa*, which shifts from autotrophic to heterotrophic nutrition between summer to winter^57^. Interestingly the symbionts’ TP was also relatively high in this study (symbiont TP: deep = 2.25 ± 0.16, shallow = 2.00 ± 0.15), and similar to that of fed *S. pistillata* kept in darkness^54^. The high TP of *O. patagonica*’s algal symbionts suggests that heterotrophically-acquired amino acids were transferred from the host to the symbionts in situ*.* Such transfer of heterotrophic products was previously observed in the Mediterranean species *C. caespitosa* in winter^57^. This notion is also supported by the ex-situ labelled *Artemia* experiment, in which symbionts had higher assimilation rates of heterotrophically-acquired carbon and equivalent assimilation of heterotrophically-acquired nitrogen compared to the host (per unit surface area, Fig. 4). These results show that a burst of plankton, in the form of labelled *Artemia*, provided five-times more carbon and ten-times more nitrogen to the symbionts than dissolved forms of carbon and nitrogen. In addition, symbionts of *O. patagonica* had 10 times lower autotrophic assimilation of dissolved inorganic carbon compared to symbionts of *C. caespitosa*^27^ and shallow tropical coral species^58^. Low autotrophic potential likely results from adaption to low in situ irradiance. Furthermore, heterotrophic dominance likely sustains *O. patagonica* during seasonal bleaching known of this population^8,29^. Altogether, our observations add support to the notion that *O. patagonica* at most facultatively uses Symbiodiniaceae for translocated carbon and suggests that these symbionts have additional obligate role(s) in coral host physiology that remain to be identified.

The similar *δ*^13^C values of the essential amino acids between host and symbionts further suggest that both partners share the same carbon source, in agreement with the latest observations performed on tropical shallow corals^53,59,60^. In addition, there was no difference in the carbon signatures of colonies between different depths. This contrasts to other studies that found differences between colonies at different sites of similar depth^61^, with different symbiont identity^60^, or corals at different light intensities (depth proxy)^59^. The lack of differentiation in the present study may be due to our additional findings that colonies were dominantly heterotrophic, grew at the same light intensity, and possessed the same symbiont species.

Despite all colonies having the same TP and carbon source (Fig. 5), different *δ*^15^N values in the host and symbiont phenylalanine between shallow and deep (Fig. 6A). As this is an essential amino acid, theoretically not affected by trophic discrimination, this result suggests that the corals prey on different food sources at each depth. Such difference may be due to the contrasting orientation of the corals upon the substrate at the two sites (Fig. 3A,D). Deep colonies grew on horizontal substrates, often with vertical extensions of the skeleton, and likely passively receive sinking organic matter. In contrast, shallow colonies were found in small caves typically orientated on vertical surfaces or upside-down on cave ceilings and therefore would need to actively capture prey. Such different trophic regimes can also be observed through the different assimilation rates of labelled *Artemia* (Fig. 4) in that deep colonies were isotopically depleted compared to the shallow ones, suggesting that they were not “primed for” predation. On the contrary, shallow colonies quickly profited from the sudden food availability, when *Artemia* prey was delivered. Such capacity to exploit a sudden abundance of food is known in anthozoans^62^.

Fine et al.^34^ proposed that *O. patagonica* invaded the Mediterranean within anthropogenic time scales, expanding its range from west to east. However, later genetic testing did not support this suggestion^37^. Instead, the large genetic diversity of this species within the Mediterranean, together with the distinction from western North Atlantic populations suggests that this species has existed in the Mediterranean for the last ~ 5 million years, perhaps in lower abundance or in more cryptic spaces, but has recently increased in abundance across the Mediterranean in part due to environmental change^37^. We suggest a similar range expansion and proliferation of *O. patagonica* has occurred in deeper sites of the Israeli Mediterranean to avoid the heat stress that with other factors can cause bleaching and death of the colony^63^. It was already evident in *C. caespitosa* that, although it prefers the shallow and well-lit environment, there is a thermally induced mortality at the shallows compared to the deep colonies^6^. This expansion was probably facilitated by the nature of the eastern Mediterranean population to live in low light environments, in contrast to the western Mediterranean where they live exposed to high light^14^. Though specimens from only one ‘deep’ site (Leonid Wreck) were included in this study, colonies were also observed in the same period at two other monitoring sites below 25 m water depth on the Israeli coastline, namely Ashdod and Ashkelon, as well as by SCUBA divers on a second wreck in Haifa Bay (Scirè submarine); these sites are spread over 150 km of coastline. Since 2014, MKMRS LTER has monitored eight sites deeper than 25 m along the Israeli coast twice a year. All sites are surveyed with the same methodology and therefore we are confident to ascertain that the proliferation of *O. patagonica* at these sites is novel and recent. The Israeli continental shelf has a very moderate slope with a minimum distance of 1 km between shallow and mesophotic sites, separated by sediments. Therefore, we propose that this migration was through the recruitment of sexually produced larvae to the new site. Additionally, *O. patagonica* colonies are still sparsely distributed at mesophotic depths and have not yet been detected in repeated photo-quadrat surveys (for methodological details and data download: https://med-lter.haifa.ac.il/index.php/en/data-base). Previous work has shown that, regardless of the prevailing thermal regime, *O. patagonica* has a fixed thermal threshold of 32 °C^7^. Therefore, we suggest that the rise in sea surface temperature in this region of ca. 3 °C in three decades, reaching 31 °C in the summer^36^, maybe forcing *O. patagonica* to migrate to deeper waters within the Israeli Mediterranean, which can be 1–2 °C cooler in the summer months^64^ (Fig. 1C).

Further work should seek to detail the thermal physiology of this population using thermal performance curves. As recently suggested, congeners from shallow and deep water can have a similar thermal tolerance^16^. Similar data in *O. patagonica* will help in distinguishing the potential reason for this species' spread in deeper waters. It will also be valuable to investigate whether the deeper colonies are themselves reproductively active and, if so, have similar fecundity to shallow conspecifics. Recent works note a reduction in reproductive fecundity in other coral species with increasing depth^18,20,21^, citing reduced light and therefore reduced fixed carbon translocated from symbionts as the driver. With *O. patagonica* inhabiting low light environments in both shallow and deep waters, it will be interesting to ascertain whether reproductive output and phenology are similar between both depth zones in this species. Finally, whether the high light adapted colonies elsewhere in the Mediterranean will also migrate deeper in the future remains an open question, together with what roles phenotypic plasticity and environmental acclimation will have in such a case.

As corals undergo widespread declines in response to global climate change and local anthropogenic impacts, better understanding what underpins this species' resilience, plasticity, and opportunistic nature may have applications for improved management and conservation success for corals worldwide.

## Conclusions

Until 2019, this species was reported up to a maximum of 15 m depth in the Mediterranean Sea; on the Israeli coastline, it was found predominantly in caves, overhangs, or other low light environments. We report novel observations of the Mediterranean scleractinian coral *Oculina patagonica* growing at a water depth of up to 35 m on the Israeli coastline. We found that colonies from both shallow water (1–3 m depth) and deep water (> 30 m) did not have significantly different physiology, symbiont association, skeletal morphology, or TP. Corals from both environments hold a relatively high TP indicating that their energy is obtained mostly heterotrophically. We postulate that this diet strategy and prior adaption to low light intensity niches facilitated this species’ migration to deeper water. We suggest that warmer sea surface temperatures are pushing *O. patagonica* to deeper cooler waters. Further testing of this coral’s thermal response and continued monitoring of both shallow and deep populations are needed to test this hypothesis.

## Material and methods

### Coral collection

Colonies of *Oculina patagonica* were collected from the Israeli Mediterranean Sea (Fig. 1A) on 17th November 2020 using a hammer and chisel to remove them from the substrate. Colonies designated as "shallow" were collected between 1 and 3 m water depth (n colonies = 8) at Sdot-Yam (32.49243N 34.8869E, Fig. 1B). "Deep" colonies were collected at 30 m depth in Haifa Bay from the Leonid wreck (32.86975N 34.95405E) (n colonies = 5). Both sites are surveyed in the spring and fall of each year by MKMRS LTER who conduct both photo quadrat and presence/absence surveys of algae, invertebrates, and fish. In addition, long-term temperature measurements at 30 and 7 m were conducted by the Israeli School of Marine Sciences in Michmoret. Data available: http://reco.ruppin.ac.il/eng/ (Fig. 1C). At the time of sample collection, temperature and light were recorded using HOBO Pendant^®^ MX Temperature/Light Data Loggers (ONSET). The water temperature was 26 °C at both depths and both coral collection sites had a comparable light intensity at noon (2–10 µmol photons m^−2^ s^−1^). At the shallow collection site, *O. patagonica* colonies typically grow in small caves, crevasses, and overhangs in the reef that reduces the ambient light intensity similar to the 30 m depth; at the deep sites, the colonies grow on light exposed surfaces. Corals were kept in the dark and in seawater during transport to the Sdot-Yam lab where photochemistry was immediately assessed (see below). Approximately 1 cm^2^ of each genotype (colony) was preserved in DNA/RNA shield (Zymo R1100) and frozen at − 20 °C prior to DNA extraction. An additional 3 cm^2^ was flash frozen in liquid nitrogen for physiology and natural isotope analyses. The remaining colony fragments were transported live in seawater to Haifa University for further examination.

### Photochemistry

Within two hours post collection, the photochemistry of algal endosymbionts in each colony was assessed using an Imaging PAM (Walz, Germany). Corals were first dark acclimated for 15 min before a single saturation pulse was used to determine maximum quantum yield (F_V_/F_M_). A light curve with 13 incremental steps between 0 and 701 µmol photons m^−2^ s^−1^ was conducted to infer relative electron transport rate (rETR) after three minutes of actinic illumination at each step (rETR = YII*PAR). The software ‘R’ was used with a script adapted from Liberman et al.^65^ to determine relative maximal electron transport rate (rETR_MAX_), rate saturating irradiance (E_K_), photosynthetic efficiency at light limiting irradiances (α, i.e. the initial slope), and photoinhibition (β, the downward slope following rETR_MAX_ plateau).

### Physiology

The tissue of each frozen coral fragment was airbrushed into a sterile ziplock bag containing 4 mL distilled water (DW). The tissue slurry was transferred to 15 mL centrifuge tubes and electrically homogenized for 20 s before centrifugation at 500×*g* for 10 min at 4 °C. A 100 µL aliquot of supernatant was taken to determine animal host protein concentration using the Bradford assay. The absorbance of samples was read with triplicate technical replicates and protein concentration was determined against BSA standards. The remaining slurry was centrifuged at 10,000×*g* for 5 min to separate the symbiotic algae from the host tissue, which was frozen (− 80 °C) and freeze dried in preparation for natural isotope analysis. The pellet containing symbiotic algae cells was resuspended in 2 mL DW; 50 µL was taken for algal cell count and 500 µL to measure chlorophyll *a* concentration. Algal cells were imaged on a haemocytometer grid using a Nikon Eclipse (Nikon Eclipse Ti–S Inverted Microscope System) microscope to excite the chlorophyll autofluorescence with blue light. The colour threshold of images was adjusted to isolate algal cells using ImageJ software and the number of cells in four replicate corner squares were counted using the 'Analyse Particles' function. The chlorophyll aliquot was first centrifuged to remove remaining DW before extraction in 1 mL 100% acetone at 4 °C for 24 h. Sample absorbance was determined in a 96 well plate using the equations of Jeffrey and Humpfrey^66^, with path length adjusted to 0.555 cm (200 µL sample volume well^−1^). The coral surface area was measured by precisely covering the fragment in aluminium foil^67^, photographing the flattened foil, and measuring the 2D foil area using ImageJ.

### Calcification rate

Living coral fragments transported from field sites were held for 48 h in aquaria at Haifa University at temperature (25 °C) and light conditions (up to 20 µmol photons m^−2^ s^−1^) corresponding to in situ measurements, prior to calcification incubations. Calcification rate was determined using the total alkalinity (TA) anomaly method^68^. Only a subset of colonies (n = 3 colonies depth^−1^) was used for calcification rate determination due to a lack of live material and a prioritisation of other parameters. Fragments were placed in a sealed transparent cup with 0.2 µm filtered seawater and submerged in thermally constant aquaria for 3 h. Water was re-filtered post incubation and measured with an automatic alkalinity titrator (855 Robotic Titrosampler, Metrohm, Switzerland). End point alkalinity in samples was subtracted from a time zero sample i.e., no incubation. Calcification rates were calculated using equation four from Schneider and Erez^69^.

### Skeletal morphology

#### Scanning electron microscopy

Fragments of airbrushed skeletons were submerged in 3% sodium hypochlorite for an hour to remove any remaining tissue, rinsed in freshwater, and dried at 60 °C. Skeletal fragments (ca. 2 cm^2^) were mounted and vacuum coated with gold (for conductivity) prior to examination under a ZEISS Sigma™ SEM (Germany), using a SE2 detector (1–2 kV, WD = 6–7 mm) (n = 3 colonies depth^−1^). SEM images were used to measure septa width (n septa = 28 shallow, 57 deep), distance between septa (n = 30 shallow, 46 deep), and distance between spines observed on the coenosteum (n = 25 shallow, 33 deep). Replicate measurements for each structure were conducted on two or more corallites per colony.

#### Binocular microscopy

Skeletons from every colony were photographed using a light binocular microscope with a scale. Skeletal morphology of all samples was inspected following Veron^38^ for species identification. Images were also used to measure the maximal diameter of polyp calyxes, wall to wall, (n = 14–42 colony^−1^) and the spacing between neighbouring corallites measured as the distance between two columella (n = 12–42 colony^−1^) in order to compare colony morphology between depths.

Both SEM and binocular images were scaled and analysed using ImageJ software.

### DNA extraction

Genomic DNA was extracted using the Wizard^®^ Genomic DNA Purification Kit (Promega, USA). The PCR amplifications were performed using Kodaq 2X PCR MasterMix (ABM, Richmond, BC Canada) following the manufacturer's protocol with a slight modification. In brief, small fragments were placed in 1.5 mL of the manufacturer’s lysis buffer and 55 μL Proteinase K, followed by overnight incubation at 55 °C, then 750 μL of the resulting liquid was used to continue the manufacturer’s protocol. Polymerase Chain Reaction (PCR) was performed for the mitochondrial cytochrome oxidase subunit 1 region (COI) using the following primers—FOL-LDEG (forward) 5′-TCWACHAAYCATAARGAYATWGG-3′ and FOL-HDEG (reverse) 5′-TCWACHAAYCATAARGAYATWGG-3′ (modified from^70^). In addition, the internal transcribed spacer (ITS2) region of *Symbiodiniaceae* rDNA was amplified using *Symbiodiniaceae*-specific primers CS1F (forward) 5′-ACACTGACGACATGGTTCTACATGTGAATTGCAGAACTCCGTG-3′ and CS2R (reverse) 5′-TACGGTAGCAGAGACTTGGTCTTACTTATATGCTTAAATTCRGCGG-3′ taken from Arif et al.^71^. PCR products were analysed by electrophoresis on a 1% agarose gel and cleaned using Promega Wizard R^®^ SV Gel and PCR Clean-Up System following manufacturer protocols. The host COI region was sequenced by the Sanger sequencing method using the ABI 3730xl DNA Analyser while *Symbiodiniaceae* ITS2 was sequenced on the Illumina Miseq using a v2-500 cycle kit to generate 250 × 2, paired-end reads. *Symbiodiniaceae* ITS2 data was demultiplexed by the Illumina software, and the demultiplexed fastq files were further analysed. The resulting COI and ITS sequences were aligned with ClustalW^72^ to create a consensus sequence, which was blasted in NCBI's GenBank for species identification. In addition, paired forward and reverse fastq.gz files were submitted to SymPortal^73^ to assess the relative dominance of the more abundant symbiont clades in samples; a standardised quality control of sequences is completed as part of the submission. The evolutionary history of the coral host species was inferred using the Neighbour-Joining method and evolutionary distances were computed using the Maximum Composite Likelihood method (units: number of base substitutions per site) using the software MEGA.

### Bulk isotope analysis

Carbon and nitrogen assimilation in the coral host tissue and algal symbionts via heterotrophy was assessed. To do this, the microalgae *Dunaliella* sp. was first grown in a Conway medium enriched with 2 mmol L^−1^ of NaH^13^CO_3_ (98 atom% ^13^C, cat. no. 372382, Sigma-Aldrich, St Louis, MO, USA) and 1 mmol L^−1^ of ^15^NH_4_Cl (98 atom% ^15^N, cat. no. 299251, Sigma-Aldrich). Two day old *Artemia salina* nauplii were grown for two days within the culture and fed upon the ^15^N/^13^C labeled microalgae. *A. salina* were then isolated by filtration (20 µm mesh), divided to equal portions, and frozen at − 20 °C. Each portion corresponded to 53.3 ± 0.8 mg dry weight (or 300 *A. salina* L^−1^) with 290 ± 9 μg heterotrophic carbon mg^−1^ (HC) and 70 ± 2 μg heterotrophic nitrogen mg^−1^ (HN) (C:N ratio = 4.2).

Similarly, we assessed the autotrophic uptake by coral algal symbionts of dissolved inorganic nitrogen (ammonium, NH_4_+) and bicarbonate (HCO_3_^−^) using ^15^N-NH_4_+ and ^13^C-HCO_3_^−^. For this purpose, several beakers containing 200 mL filtered seawater (FSW) were prepared and enriched with 3 µM NH_4_^+^ and 0.3 mM HCO_3_^−^. Corals (n = 4 depth^−1^ feeding condition^−1^) were placed in an incubator at 26 °C with 90 µmol photons m^−2^ s^−1^ (closely matching the saturation irradiance of the colonies, see results) and incubated with either the labelled *A. salina* or dissolved compounds for 5 and 7 h, respectively, with regular stirring. After incubation, coral fragments were rinsed with FSW before being frozen in liquid nitrogen. Coral tissue was removed as above and separated by centrifugation to animal and algal components before freeze drying. Approximately 500 µg of lyophilized host and symbiont material were transferred to tin caps for analysis of the natural isotopic signals, ^15^N and ^13^C enrichment, as well as total carbon and nitrogen content using an Integra II isotope ratio mass spectrometer (Sercon, United Kingdom). The assimilation rates of dissolved inorganic nitrogen and carbon were calculated according to previously defined equations^74,75^, taking into account the nitrogen and carbon content as well as the nitrogen and carbon enrichment in the coral tissue or in the symbionts, compared to the natural isotopic values. Assimilation rates were expressed per hour and per skeletal surface area of the coral nubbins.

### Amino acids compound-specific stable isotope analysis (AA-CSIA)

Approximately 3 mg of lyophilized coral host tissue or symbiont was acid hydrolysed in 0.5 mL of 6 nmol HCl at 150 °C for 75 min^76^ under nitrogen atmosphere inside a 4 ml glass vial with PTFE cap. Samples were cooled to room temperature and then HCl was evaporated under a gentle stream of nitrogen. Samples were neutralized twice with 0.5 mL ultra-pure water and evaporated with a gentle stream of nitrogen. Chloroformate derivatization was used with the EZfaast amino acid analysis kit (Phenomenex Inc.), replacing reagent 6 with dichloromethane as a solvent. The amino acids were separated on a Zebron ZB-50 column (30 m, 0.25 mm, and 0.25 µm) on a Thermo Scientific Trace 1300 Gas Chromatograph with helium as a carrier gas at a constant flow of 1.5 mL min^−1^. For carbon analysis, 1.5 µL was injected in split mode (1:15) at 250 °C and 2 µL was injected in split mode (1:5) at 250 °C for nitrogen analysis. GC conditions were set to optimised peak separation for the desired amino acids as follows: initial temperature 110 °C ramped to 240 °C at 8 °C per min and then ramped to 320 °C at 20 C per min and held for 2.5 min. The separated amino acids were split on a MicroChannel device into two direction flows: one toward a Thermo Scientific ISQ quadruple for amino acid identification and the second toward a Thermo Scientific Delta V advantage for carbon and nitrogen isotope analysis. The ISQ condition was set to: transfer line 310 °C, ion source 240 °C and scan range from 43 to 450 m/z mass range. To define the isotopic ratio of carbon and nitrogen the separated amino acids were combusted in a Thermo Scientific GC Isolink II at 1000 °C for CO_2_ and N_2_. Before entering the Delta V for the N_2_ analysis, the sample went through a liquid nitrogen cold trap to freeze down all other gases. From each sample, technical duplicates were injected for carbon and triplicates for nitrogen.

Stable isotope ratios were expressed in standard δ notation where the standard for carbon was Vienna PeeDee Belemnite (VPDB) and for nitrogen atmospheric N_2_ (air). Separated amino acids were purchased from Sigma Aldrich and analysed at the Geological Survey of Israel on an elemental analyser (1112 Flash EA, Thermo) interfaced with isotope ratio mass spectrometer (IRMS, Delta V Plus, Thermo). To extend the nitrogen isotopic range, two certified amino acids (Alanine + 43.25‰ and Valine + 30.19‰ δ^15^N) were purchased from Arndt Schimmelmann, (Indiana University). The standard we used contains seven amino acids of known isotopic ratio with isotopic range for nitrogen of − 6.69 to + 43.25‰ δ^15^N. To account for the carbon incorporated during the derivatization process, the following correction factor for each amino acid was used:

$${\text{n}}_{{{\text{cd}}}} \delta^{13} {\text{C}}_{{{\text{cd}}}} = {\text{n}}_{{\text{c}}} \delta^{13} {\text{C}}_{{\text{c}}} + {\text{n}}_{{\text{d}}} \delta^{13} C_{{{\text{dcorr}}}}$$

where n is the number of moles of carbon, Cc the amino acid (AA) of interest, Ccd the derivatized compound, and Cdcorr the empirically determined correction factor^77^. The standard AA was used to set Cdcorr for later calculation of the isotopic ratio of the sample. Each standard was injected three times after the combustion reactor oxidation for carbon and three more times for nitrogen to allow for drift correction and injected again after a maximum of 18 injections. Since AAs differ in the presence of heteroatoms and functional groups, which may lead to different combustion efficiencies, an average of the standard injection from the beginning and the end of the sequence was used. For each sequence of nitrogen, a correction factor was applied based on the linear regression equation of the ratio between the known AA isotopic ratio and the acquired result for the sequence. Since there is no addition of exogenous atoms of nitrogen in the derivatization process there is no need for correction per AA.

Trophic level or trophic position (TP) was calculated according to Martinez et al.^52^. TP measures the position of a species in a food web. Primary producers such as phytoplankton or plants (i.e., autotrophs) have a TP of 1, while the TP of primary consumers, which eat primary producers (i.e., heterotrophs) is 2. Secondary consumers have a TP greater than 2. In mixotrophic organisms, such as corals, which live in symbiosis with photosynthetic dinoflagellates, a TP around 1 indicates that the association is mostly autotrophic (relying on the photosynthates acquired by the dinoflagellates), while a higher than 1 indicates a heterotrophic input^54,59^.

TP was calculated according to the following equation:

$${\text{TP}} = \left( {\left( {\delta^{{{15}}} {\text{NGlu}}{-}\delta^{{{15}}} {\text{NPhe}}{-}\beta } \right)/{\text{TDF}}_{{{\text{AA}}}} } \right) + {1}.$$

The constant, β, is the difference between the δ^15^N values of glutamic-acid and phenylalanine AAs in primary producers (TP 1). The trophic discrimination factor (TDF_AA_) is the average δ^15^N enrichment relative to source AAs per TP. The constants β = − 0.36 and TDF_AA_ = 4.54 were used since they provided the best fit to the derivatization method^52^.

### Statistical analysis

Data analysis and graphics were complied with the software R v4.0.3^78^. Data from each parameter were tested for homogeneity of variance within each collection depth using Levene’s Test (*leveneTest*::car)^79^ and normal distribution was visualized graphically (*hist*::graphics) and statistically tested (*shapiro.test*::stats)^78^. One extreme outlier data point was removed from the photophysiological parameter β/beta (deep). Statistical differences in the assessed parameters between shallow and deep collection depths were examined using a Student's T-test (*t.test*::stats)^78^. In the cases that parametric assumptions were not met by the data (Table 1), a Kruskal Wallis test was implemented (*kruskal.test*::stats)^78^. Boxplot graphics were constructed (*ggplot*::ggplot2) and combined (*plot_grid*::cowplot) in R^80,81^. A principal component analysis (PCA) and analysis of similarities (ANOSIM) was conducted based on the Euclidean distance resemblance matrix as a response variable. Data cited in the text are mean ± standard error.
